# Prevalence of a Composite Outcome to Capture Child and Liver Health One Year After Pediatric Liver Transplantation in the Starzl Network

**DOI:** 10.1111/petr.70402

**Published:** 2026-07-11

**Authors:** Frank DiPaola, Amy Feldman, Vicky Ng, Swati Antala, John Bucuvalas, Noelle Ebel, Nitika Gupta, Jennifer Halma, Rohit Kohli, Daniel Leung, Steven Lobritto, Anna Peters, James Squires, Emily Perito

**Affiliations:** ^1^ Division of Pediatric Gastroenterology, Department of Pediatrics University of Virginia Charlottesville Virginia USA; ^2^ Section of Pediatric Gastroenterology, Hepatology and Nutrition, Digestive Health Institute University of Colorado School of Medicine and Children's Hospital Colorado Aurora Colorado USA; ^3^ Division of Gastroenterology, Hepatology and Nutrition, Hospital for Sick Children University of Toronto Toronto Ontario Canada; ^4^ Mount Sinai Kravis Children's Hospital and Recanati/Miller Transplantation Institute Mount Sinai Health System New York New York USA; ^5^ Stanford University Palo Alto California USA; ^6^ Children's Healthcare of Atlanta and Emory University School of Medicine Atlanta Georgia USA; ^7^ Department of Pediatrics University of Missouri‐Kansas City School of Medicine Kansas USA; ^8^ Department of Pediatrics, Division of Gastroenterology, Hepatology and Nutrition Children's Hospital Los Angeles Los Angeles California USA; ^9^ Division of Pediatric Gastroenterology, Hepatology and Nutrition, Department of Pediatrics Baylor College of Medicine Houston Texas USA; ^10^ Columbia University Medical Center, Morgan Stanley Children's Hospital of New York New York New York USA; ^11^ Department of Pediatrics University of Cincinnati Cincinnati Ohio USA; ^12^ Department of Pediatrics University of Pittsburgh School of Medicine and UPMC Children's Hospital of Pittsburgh Pittsburgh Pennsylvania USA; ^13^ Department of Pediatrics University of California San Francisco San Francisco California USA

**Keywords:** comparative effective trials, composite outcome, immunosuppression, patient‐centered outcomes, pediatric liver transplantation

## Abstract

**Background:**

The Starzl Network for Excellence in Pediatric Transplantation, a learning health network focused on pediatric liver transplantation, identified optimizing immunosuppression as a priority challenge. There remains significant variability in immunosuppression practices across transplant centers and a need for comparative effectiveness research to clarify best practices. As a prelude, we aimed to develop a composite outcome that focuses on immunosuppression‐related morbidity and to characterize its baseline prevalence at 1 year post‐liver transplant.

**Methods:**

We developed a composite transplant primary outcome, based on a previously described “ideal outcome” definition utilized by the Society of Pediatric Liver Transplantation registry studies, with components reflecting liver and child health. We used retrospective data from Starzl Network centers to calculate the prevalence of achieving the composite outcome among children who underwent a liver transplant in 2023.

**Results:**

In 2023, 19 participating centers transplanted a total of 267 children. Of the 250 with complete data available, 46% were < 2 years of age at transplant, 25% were 2–8 years, 22% were > 8 years, and 7% were missing age data. Seventy‐eight children (31%) achieved the composite outcome at 1 year, including 35% of < 2‐year‐olds, 32% of 2–8 year‐olds, 29% of > 8 year‐olds, and 11% without age available. The most common failed composite outcome criterion was having ALT > 50 IU/L at 1 year.

**Conclusions:**

Only 31% of pediatric liver transplant recipients achieved the composite outcome by 1 year. Our network will aim to develop evidence‐based best practices to increase achievement of the composite outcome by “right‐sizing” immunosuppression for each child.

**Abbreviations**
ALT: alanine aminotransferase
CER: comparative effectiveness research
C‐TPO: composite transplant primary outcome
eGFR: estimated glomerular filtration rate
GGT: gamma glutamyltransferase
IS: immunosuppression
LT: liver transplant
PCOR: patient‐centered outcomes research
PTLD: post‐transplant lymphoproliferative disorder
SNEPT: Starzl Network for Excellence in Pediatric Transplantation
SPLIT: Society for Pediatric Liver Transplantation

## 1 Introduction

The Starzl Network for Excellence in Pediatric Transplantation (SNEPT) is a learning health network that leverages provider–patient partner collaboration and patient‐centered outcomes research (PCOR) to address major challenges facing pediatric liver transplantation (LT) [1]. Transplant recipients and families actively collaborate in project prioritization and design to ensure that outcomes improvement efforts are meaningful to all [2].

In 2018, SNEPT chose optimization of immunosuppression (IS) as a network priority. Intercenter variability in IS practices and insufficient detail on post‐LT outcomes data were identified as major barriers to evidence‐based optimization of IS for children after LT. Recently published data demonstrate the significant variability in IS practices among the 16 original SNEPT centers (Table S1; adapted from Tables S1 and S2 in the article by Antala et al. [3]). The Society for Pediatric Liver Transplantation (SPLIT) also recently identified reducing redundant variability and optimizing perioperative IS management as a high‐priority research area, clarifying the need for multicenter, prospective comparative effectiveness research (CER) to facilitate the development of evidence‐based best practices for IS [1, 2, 4].

A major historical limitation of IS trials has been use of either rejection ***or*** IS‐related adverse effects as their primary outcome. However, IS management always requires balancing the risks of excessive with those of inadequate IS. CER intended to guide IS management in real‐world settings would ideally use a primary outcome that captures both the IS risks and benefits. Previous pediatric LT registry analyses defined a composite “ideal outcome” based on Porter's 3‐tier outcome hierarchy [5]. Recent engagement with patient partners and pediatric LT providers further clarified characteristics of a meaningful CER outcome [2].

As groundwork for patient‐centered CER on post‐LT IS, we developed a composite outcome that focuses on IS‐related morbidity and characterized its baseline prevalence at 1 year post‐LT.

## 2 Methods

Potential components of our “composite transplant primary outcome” (C‐TPO) were selected from the previously defined “ideal outcome” utilized by SPLIT in its registry studies. The initial “ideal outcome” included 13 components that addressed allograft sustainability (Tier 1), absence of IS‐induced comorbidity (Tiers 2 and3), treatment burden and longer term outcomes [5, 6, 7]. (personal communication, Vicky Ng). The C‐TPO was refined to focus on (1) components directly related to IS and (2) following stakeholder priorities identified in the SNEPT PARTNER PCOR Roadmap (https://partner.starzlnetwork.org/) and evaluated using our PCOR Outcomes Scorecard (https://partner.starzlnetwork.org/design‐the‐project/) [2].

SNEPT centers extracted de‐identified retrospective data from the electronic medical record on transplant recipients in 2023. For feasibility, data collection was limited to age at transplant (< 2 years, 2–8 years, > 8 years), yes/no status of binary C‐TPO components, and key laboratory values, including alanine aminotransferase (ALT), gamma glutamyl transferase (GGT), and estimated glomerular filtration rate (eGFR). Data within a 2‐month window of 1 year post‐LT were accepted. Achieving C‐TPO was defined as meeting all criteria. We calculated the prevalence of achieving C‐TPO and each individual C‐TPO criteria.

This analysis was certified Not Human Subjects Research by the UCSF Committee on Human Research (CHR) because only de‐identified data were collected; it was exempt from CHR approval.

## 3 Results

The C‐TPO (Table 1) incorporates universally reported transplant outcomes, specifically patient and graft survival, minimal to no graft inflammation as defined by ALT and GGT < 50 IU/L, avoidance of posttransplant lymphoproliferative disorder (PTLD), as well as criteria identified as important to recipients and families including avoidance of renal impairment and reduction of medication burden.

**TABLE 1:** Composite‐transplant primary outcome (C‐TPO).

Health of liver	Health of child
No graft failure Minimal/no liver inflammation: ALT< 50 IU/L + GGT < 50 IU/L	Alive No post‐transplant lymphoproliferative disease (PTLD) Off corticosteroids (or lowest intended maintenance dose) No impaired kidney function (GFR > 75% normal)† No medications for hypertension^b^, diabetes^a^, ^b^, or seizures^a^

Abbreviations: ALT, alanine aminotransferase; GGT, gamma glutamyl transferase.

^a^ Side effect of tacrolimus.

^b^ Side effect of corticosteroids.

Data were collected from 19 SNEPT centers on a total of 267 children transplanted in 2023. Complete data on all C‐TPO criteria were available for 94% of recipients. Of the 17 children with incomplete data to calculate C‐TPO, 12 were alive at 1 year, 10 were reported as no re‐transplant, and 7 were without PTLD.

**FIGURE 1:**
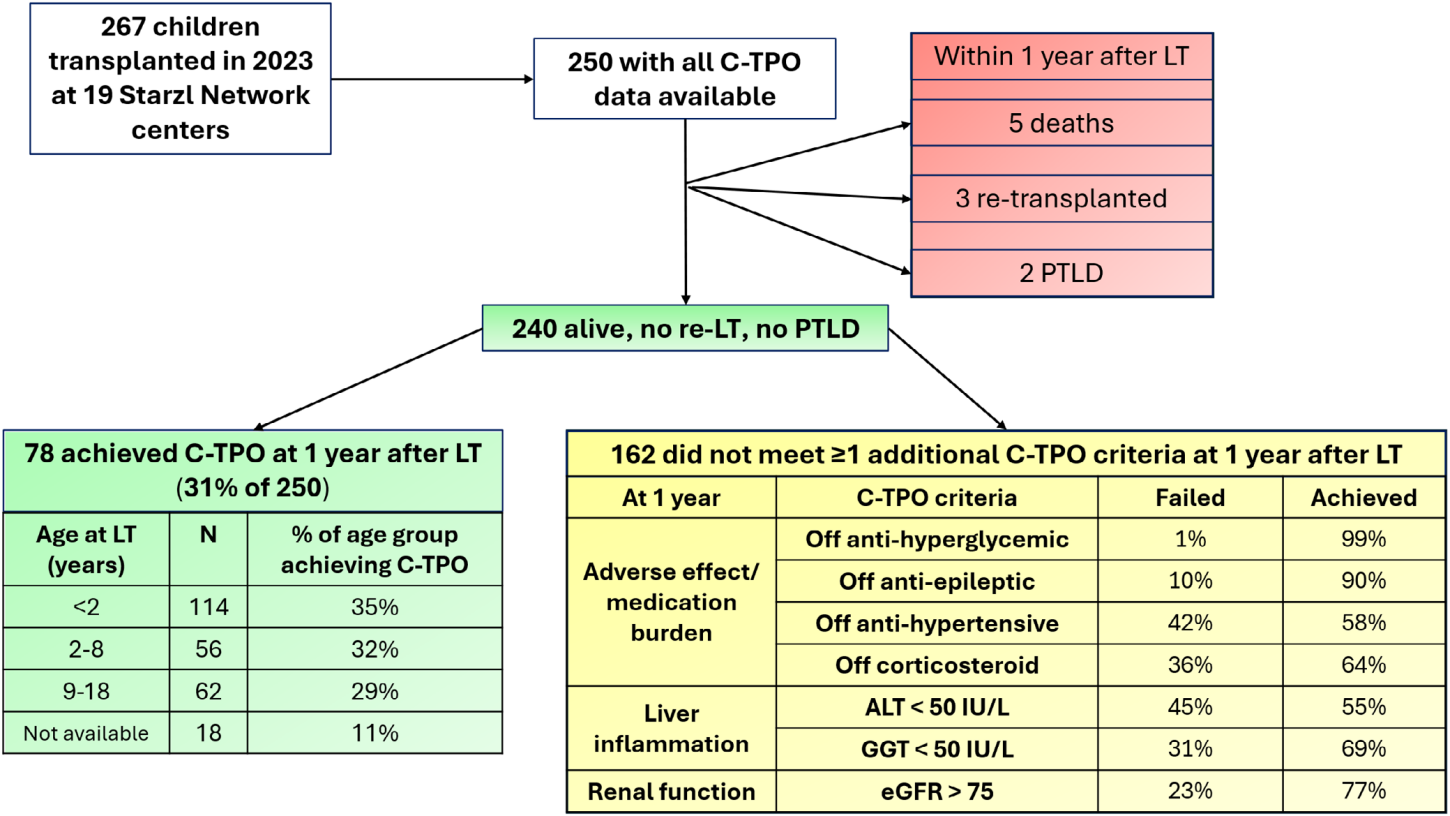
Prevalence of the composite transplant primary outcome (C‐TPO) and its components in pediatric liver transplant recipients, 1 year posttransplant.

Of the 250 children with complete C‐TPO data available, 46% were < 2 years of age at transplant, 25% were 2–8 years, 22% were > 8 years, and 7% were missing age data. Seventy‐eight children (31%) achieved C‐TPO at 1 year, including 35% of < 2‐year‐olds, 32% of 2–8 year‐olds, 29% of > 8 year‐olds, and 11% without age available. (Figure 1) 98% of children were alive at 1 year (5 deaths). Only 1% of survivors required re‐transplant and 1% developed PTLD before 1 year. Only two children still required diabetes medication at 1 year; both were on steroids. The most common failed C‐TPO criteria were still requiring anti‐hypertensive medications at 1 year, still being on corticosteroids, and having ALT> 50 IU/L at 1 year. Among children still on steroids at 1 year (*n* = 59), 39% required anti‐hypertensives (*n* = 59; vs. 25% not on steroids) and 39% had ALT> 50 IU/L (vs. 26% not on steroids).

## 4 Discussion

Our refined C‐TPO focuses on IS‐related outcomes and integrates broad stakeholder input on meaningful outcomes for patients. Utilizing a composite outcome that integrates both IS efficacy and safety mirrors recent trials in pediatric solid organ transplant [8, 9] and is supported by regulatory body and expert guidance on meaningful outcomes in IS research [10, 11].

Herein, only 31% of pediatric LT recipients achieved the C‐TPO by 1 year. Graft dysfunction and persistent corticosteroids were common barriers to achieving C‐TPO, as were adverse effects of IS including hypertension and renal insufficiency. This is a clear opportunity for IS optimization to support graft and patient health, minimize IS‐related side effects, and reduce medication burden.

Future research will seek to validate C‐TPO as a predictor of longer term outcomes and utilize SNEPT to compare IS treatment strategies. C‐TPO refinement, for example, by weighing critical components more heavily, may also be helpful in optimizing its utility. Although not every child will achieve C‐TPO by 1 year, we aim to develop evidence‐based best practices to increase the percentage of children achieving C‐TPO by “right‐sizing” IS for each child [1].

## Funding

This work was supported by Citrone Family and Citrone 33.

## Supporting information


**Table S1:** Initial immunosuppression after isolated pediatric liver transplant at 16 Starzl Network Centers (adapted from Antala et al. Liver Transpl 2026.)

## Data Availability

The data that support the findings of this study are available from the corresponding author upon reasonable request.
