# Supplementary material for: Prevalence of a Composite Outcome to Capture Child and Liver Health One Year After Pediatric Liver Transplantation in the Starzl Network
Source: Pediatr Transplant. 2026 Jul 11;30(7):e70402. doi: 10.1111/petr.70402 (PMC13355088; doi:10.1111/petr.70402)
Supplement: Supplementary file 1 — Table S1: Initial immunosuppression after isolated pediatric liver transplant at 16 Starzl Network Centers (adapted from Antala et al. Liver Transpl 2026.) [file PETR-30-e70402-s001.docx]

Supplemental Table 1: Initial immunosuppression after isolated pediatric liver transplant at 16 Starzl Network Centers (Adapted from: Antala et al. Liver Transpl 2026.)

|  | **Standard for most LT**  **(n of centers)** | **Special populations only**  **(n of centers)** | **Dosing strategies** | **Never/rarely used for isolated LT**  **(n of centers)** |
| --- | --- | --- | --- | --- |
| **Basiliximab** | 3 | 10   - Renal-sparing (8) - Re-LT (1) - Tumor, ALF, <4 years of age (1) - When using MMF (1) | **Standard:** doses on POD0 and POD4, 10mg if child is <35kg and 20mg if ≥35kg on POD0 (12 centers)  **Weight-based dosing:** 1 center | 3 |
| **Thymoglobulin** |  | 8   - Re-LT or renal-sparing (3) - Stable metabolic or chronic liver disease (3) - ABO incompatible (1) | **Standard** 1-3 doses total, with maximum total dose 5-7 mg/kg (5 centers)  **Monitored dosing** based on CD3 or lymphocyte counts, 1-5 doses given with maximum 5-7.5 mg/kg total (3 centers) | 8 |
| **Mycophenolate mofetil (MMF)** | 5* | 6   - Autoimmune hepatitis (6) - ABO incompatible (1) - Renal-sparing (1) - When using basiliximab (1) | **Initiated on post-operative day (POD):**   - POD 0-1: 8 centers - POD 1-2: 1 center (for AIH LTs) - POD 0-30: 1 center (for AIH LTs)   **Starting dose:**   - 7.5-10 mg/kg/dose: 3 centers - 10-15 mg/kg/dose: 4 centers - 20-25 mg/kg/dose: 2 centers - Dosing based on body surface area: 2 centers   **Anticipate stopping by post-operative month (POM):**   - <12m: 2 centers - 12m: 3 centers - > 12m, or continue maintenance after LT for AIH: 5 centers | 5 |
| **Corticosteroids** | 16 | 0 | Dosing and taper trajectory varies widely† | 0 |
| **Tacrolimus** | 16 | 0 | **Initiated on:**   - POD 0-1: 15 centers - POD 2: 1 center - (POD3: 1 center, when used with thymoglobulin)   **Starting dose:**   - 0.05 – 0.1 mg/kg/dose: 14 centers - 0.1 – 0.3 mg/kg/dose: 2 centers   **Goal trough used by majority of centers, POM:** †   - POM 0-1: 10-12 - POM 1-2: 8-12 - POM 3-6: 6-10 - POM 7-12: 5-8 | 0 |

* One center uses routinely for isolated pediatric LT except if transplant indication is tumor.

† See for additional detail: Antala S, Halma J, Batsis I, et al. Development of multicenter consensus care plans for immunosuppression after pediatric liver transplant: Reducing variability, creating comparative efficacy opportunities. Liver Transpl. Published online June 22, 2026. Doi: 10.1097/LVT.0000000000000933.
